# Lay advisor interventions for hypertension outcomes: A Systematic Review, Meta-analysis and a RE-AIM evaluation

**DOI:** 10.3389/fmed.2024.1305190

**Published:** 2024-05-20

**Authors:** Sonal J. Patil, Vishwa Bhayani, Yilin Yoshida, Leila Bushweller, Eno-Obong Udoh, Irina Todorov, Robert Saper, Kurt C. Stange, Shari Bolen

**Affiliations:** ^1^Center for Health Equity, Engagement, Education, and Research (CHEEER), Department of Family Medicine, The MetroHealth Campus of Case Western Reserve University, Cleveland, OH, United States; ^2^Department of Public Health, University of Missouri, Columbia, MO, United States; ^3^Department of Medicine, School of Medicine, Tulane University, New Orleans, LA, United States; ^4^Cleveland Clinic Lerner College of Medicine of Case Western Reserve University, Cleveland, OH, United States; ^5^Department of Wellness and Preventive Medicine, Primary Care Institute, Cleveland Clinic, Cleveland, OH, United States; ^6^Center for Community Health Integration, Case Western Reserve University School of Medicine, Cleveland, OH, United States; ^7^Department of Medicine, Center for Health Care Research and Policy, The MetroHealth Campus of Case Western Reserve University, Cleveland, OH, United States

**Keywords:** allied health personnel, lay advisors, community health workers, health care systems, implementation sciences, hypertension, RE-AIM

## Abstract

**Introduction:**

Lay advisor interventions improve hypertension outcomes; however, the added benefits and relevant factors for their widespread implementation into health systems are unknown. We performed a systematic review to: (1) summarize the benefits of adding lay advisors to interventions on hypertension outcomes, and (2) summarize factors associated with successful implementation in health systems using the Reach, Effectiveness, Adoption, Implementation, Maintenance (RE-AIM) framework.

**Methods:**

We systematically searched several databases, including Ovid MEDLINE, CINAHL, PsycINFO from January 1981 to May 2023. All study designs of interventions delivered solely by lay advisors for adults with hypertension were eligible. If both arms received the lay advisor intervention, the study arm with lower intensity was assigned as the low-intensity intervention.

**Results:**

We included 41 articles, of which 22 were RCTs, from 7,267 screened citations. Studies predominantly included socially disadvantaged populations. Meta-analysis (9 RCTs; *n* = 4,220) of eligible lay advisor interventions reporting outcomes showed improved systolic blood pressure (BP) [−3.72 mm Hg (CI –6.1 to −1.3; *I*^2^ 88%)], and diastolic BP [−1.7 mm Hg (CI −1 to −0.9; *I^2^* 7%)] compared to control group. Pooled effect from six RCTs (*n* = 3,277) comparing high-intensity with low-intensity lay advisor interventions showed improved systolic BP of −3.6 mm Hg (CI –6.7 to −0.5; *I*^2^ 82.7%) and improved diastolic BP of −2.1 mm Hg (CI –3.7 to −0.4; *I*^2^ 70.9%) with high-intensity interventions. No significant difference in pooled odds of hypertension control was noted between lay advisor intervention and control groups, or between high-intensity and low-intensity intervention groups. Most studies used multicomponent interventions with no stepped care elements or reporting of efficacious components. Indicators of external validity (adoption, implementation, maintenance) were infrequently reported.

**Discussion:**

Lay advisor interventions improve hypertension outcomes, with high intensity interventions having a greater impact. Further studies need to identify successful intervention and implementation factors of multicomponent interventions for stepped upscaling within healthcare system settings as well as factors used to help sustain interventions.

## 1 Introduction

Hypertension is the leading risk factor for heart disease, and 31.3% of adults worldwide have hypertension (1, 2). It is estimated that only 13.8% of patients with hypertension globally achieve hypertension control (2). Traditional clinic-based care has not successfully improved hypertension control rates, which are worse in underserved communities (1). Community-based support improves outcomes in socially disadvantaged populations, especially when delivered by lay advisors who belong to the same social groups (3). Prior reviews of lay health advisors and community health workers (CHWs) have shown improved blood pressure and hypertension control (4–6). These reviews have been limited by including studies that evaluated lay advisor interventions with team-based care or additional health professional interventions and infrequent inclusion of broader community-based lay advisors such as barbers and faith-based lay advisors. Most health systems do not have the resources and staff to include multilevel interventions as reimbursement structure for team-based care is unclear, and it is difficult to know which level of intervention intensity can improve outcomes and in which contexts. The Community Preventive Services Task Force’s (CPSTF) systematic review of CHW interventions for heart disease and stroke prevention reported an evidence gap in incremental effectiveness of CHW interventions (7). Therefore, there is a need to identify the sole benefit of adding lay advisors to improve their adoption into routine healthcare teams, assess their generalizability or external validity, and understand the level of intensity and context needed to have an impact on blood pressure.

Thus, we conducted a systematic review which aims to assess the additional benefit of lay advisor interventions (including varying intensity levels) on hypertension outcomes from a health system perspective. We defined lay advisor interventions as those provided by anyone who does not have a health professional degree, including CHWs, health coaches, hairdressers, and faith-based workers. We aim to summarize reported factors that may inform decisions on implementation choices in clinical settings using the Reach, Effectiveness, Adoption, Implementation, and Maintenance (RE-AIM) framework, which is useful for assessing internal and external validity and context of interventions (8).

## 2 Methods

The PRISMA statement was used to report the findings of this systematic review (9).

### 2.1 Search strategy and study selection

(See Supplementary material S1 for the detailed search strategy for the Ovid MEDLINE database)

### 2.2 Data sources

Librarians with expertise in screening citations for systematic reviews searched English language articles from 1981 through May 2023, using Ovid MEDLINE, Cochrane Central Register of Controlled Trials, Cochrane Database of Systematic Reviews, CINAHL, PsycINFO, Scopus, World Health Organization International Clinical Trials Registry Platform (WHO ICTRP), ClinicalTrials.gov, and Sociological Abstracts. We reviewed references in published reviews for any additional articles. Two reviewers (SJP and VB) independently screened citations and confirmed the final included studies.

### 2.3 Search terms

Groups of search terms included keywords for (1): lay lead, peer, community health worker, promotora, expert patient, barber, hairdresser, volunteer aide, faith-based, and (2); hypertension, high blood pressure, blood pressure.

#### 2.3.1 Population and setting

Randomized and non-randomized studies published in English where the lay advisor intervention was evaluated as a sole additional intervention in adults with hypertension were included. As this review is designed for upscaling lay advisor interventions for hypertension care from a health system perspective, we did not include population-level screening studies that excluded adults with hypertension or population-level studies that did not report outcomes for the proportion of individuals diagnosed with hypertension. We excluded studies focused on pregnancy related hypertension disorders (preeclampsia, gestational hypertension).

#### 2.3.2 Intervention

We defined lay advisor interventions as those including navigation, education, or support provided by anyone who does not have a health professional degree, as they typically belong to the same social groups as study participants (10). Common lay advisor interventions include *promotoras*, health coaches, peer supporters, faith-based workers, hairdressers, and community health workers. We excluded studies that included additional health professional intervention, including physician education or intervention components, as it is typically uncompensated time. We excluded studies of blood pressure screenings in the community or health insurance linkages where patients with hypertension were excluded or there was no follow-up information on the group of patients with confirmed clinical diagnosis of hypertension. If both arms received the lay advisor intervention, the study arm with lower intensity was assigned as the low-intensity intervention. We assigned low-intervention intensity when the lay advisors delivered a synchronous intervention targeting hypertension education or management. It was not considered an intervention if the lay advisors only checked BP or collected data.

#### 2.3.3 Comparator

We included control groups where the only difference between the intervention and control group was the lay advisor delivered intervention. We included studies even if the control group received any form of low-intensity lay advisor interventions to provide insight into the incremental benefit of low-intensity versus high-intensity lay advisor interventions. Pre-post, process evaluations, and non-randomized studies were included. Studies that compared lay advisor interventions with active comparators such as health professionals or research staff were excluded.

#### 2.3.4 Outcomes

For quantitative outcomes, the primary outcome was reduction in blood pressure (BP). We included change in systolic BP and diastolic BP as our joint primary outcome. Secondary outcome was the difference in the change in the proportion of patients with controlled hypertension from baseline to post intervention between intervention and control arms. If reported, we used the proportion of patients with BP <140/90 mmHg to define controlled hypertension if the study did not explicitly state the proportion of patients with controlled hypertension (2). For RE-AIM dimension outcomes, we looked at the characteristics and presence or absence of each RE-AIM dimension component.

### 2.4 Quality assessment

Two authors assessed study quality using the Cochrane Collaboration’s risk of bias tool for RCTs (11). The primary author (S.J.P.) made final decisions where conflicts existed after reviewing all the articles independently.

### 2.5 Data extraction

Two authors independently reviewed titles, abstracts, and full articles to identify eligible studies and conflicts were resolved by joint re-review and consensus. Prior to data extraction, two authors created a codebook with all variables of interest. Two authors extracted data independently, and discrepancies were resolved by consensus between reviewers or by a third author if needed.

#### 2.5.1 Data

We extracted data on characteristics of the study setting, participants, lay advisor training and recruitment, and intervention characteristics. We defined any intervention with more than one component as multicomponent intervention. For example, if an intervention included education sessions and recurring follow-up telephone calls, it was considered a multicomponent intervention.

Quantitative values and measures of statistical variation for BP and hypertension control rates were extracted from baseline and at the end of the study. When there were multiple study arms, we included quantitative values for the two arms, where the only difference was the lay advisor-led intervention or varying levels of lay advisor intervention.

Internal and external validity indicators using RE-AIM coding and scoring: A previously published tool was used to code eligible articles on the degree to which internal and external validity indicators of the RE-AIM framework were reported (12). We looked at protocols if referenced in the main articles. Supplementary Table S1 details how each dimension and component of RE-AIM was defined and measured.

### 2.6 Statistical analysis and data synthesis

A descriptive synthesis of the study setting, participants, lay advisors, intervention components, and control group was performed and reported as a study description table. Proportions of total, RCTs, and nonRCT studies reporting each of the RE-AIM dimensions and components are reported as a table. *Quantitative synthesis:* If we had three or more eligible studies of added lay advisor intervention or varying levels of lay advisor interventions, the primary author (SJP.) performed the statistical analysis using Comprehensive Meta-analysis Software version 3 (Biostat Inc., Englewood, NJ). We adjusted sample sizes for cluster RCTs using the documented intra-cluster coefficient (ICC) (13). We used the random-effects model to compute conservative effect sizes incorporating both within-study and between-study variations. We calculated the difference in means with 95% confidence intervals, and we considered a *p*-value of <0.05 statistically significant for all analyses other than the Q statistic. A correlation coefficient of 0.5 was assumed between initial and final values. Heterogeneity among studies was evaluated using the Q statistic, with a *p*-value <0.10 indicating heterogeneity, and using *I*^2^ statistics (*I*^2^ values <40% may indicate less substantial heterogeneity and 75–100% indicates substantial heterogeneity) (14). If substantial heterogeneity existed, we planned *a priori* meta-regression if we had 20 or more studies or subgroup analysis if we had <10 studies. We identified the following study characteristics that may explain between-study variability: presence or absence of intention to treat analysis; presence or absence of home visits; settings in developed or developing countries; lay advisor training duration; study duration; and intervention components of group education, individualized intervention, or combined intervention. Publication bias was assessed with funnel plots and the Egger regression test (15). We conducted sensitivity analysis by removing one study at a time.

## 3 Results

**Figure 1:**
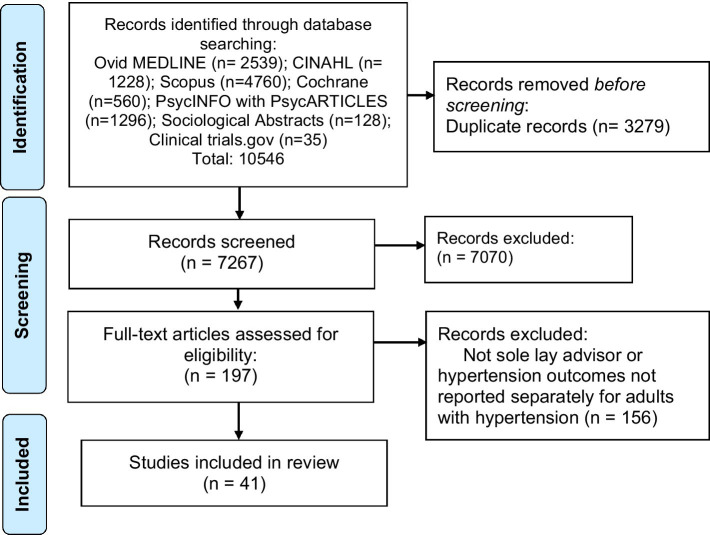
PRISMA flow diagram - identification of studies via databases and registers.

Of 7,267 unique citations, 41 studies were eligible for inclusion in our review. See PRISMA Flow Diagram. (Figure 1) All study characteristics are shown in Table 1.

**Table 1:** Study description table.

Study, year published	Study design, number of participants^a^, duration	Population, setting^b^ country urban/ rural dominant ethnicity (>40%) mean age	Interventionist, lay advisor selection and training^b^	Intervention^b,c^ components group education phone calls online community-based home visits	Comparison^b,c^
Hovell, 1984	NonRCT *n* = 20 >3 months	United States White 53 y/o	High school graduates or college students. Trained in blood pressure measurement, adherence assessment, interviewing, and counseling in three two-hour sessions (Total 6 h training) by a nurse and a psychologist.	Individual visits twice monthly: monitoring of BP and emphasis on appointment attendance and adherence to medications. Phone calls if visit was missed. Community based: no Home visits: no	None
Krieger, 1999	RCT *n* = 421 3 months	United States Urban African American predominantly and White Age mainly 40–64 year old.	Community health workers, predominantly African American and from neighborhoods similar to study subjects. 100 h of training on hypertension, cardiovascular system, risk factors for CVD, community resources, research methods, stress management, alcohol/ drugs. Certified as blood pressure measurement specialists.	Phone calls, variable frequency and duration: tracking and outreach to participants with hypertension to promote access to care and keeping appointments. Community based: No Home visits: no	Usual care
Morisky, 2002	RCT *n* = 1,367 48 months	United States Urban African American 54 y/o	Community health workers of same ethnic group assigned to each participant. 1 month training in BP measurement and monitoring, adherence counseling, barriers to care.	High intensity intervention condition: Home visits with counseling similar to low intensity intervention but also involves family/household members in supporting lifestyle and medication adherence with further opportunities to participate in discussion groups. Higher frequency of home visits done for patients with uncontrolled hypertension and low adherence. Group education: Yes in home visit intervention Community based: no Home visits: yes in home visit intervention	Low intensity intervention by CHWs: Individualized patient counseling following each clinic visit, for 5–10 min. Emphasis on lifestyle modifications, medication adherence, appointment-keeping. Assesses health literacy, barriers, social and family support.
Levine, 2003	RCT *n* = 789 40 months	United States Urban African American 43 y/o	Community health workers, local to the area, trained over 3 months in BP management, monitoring, education, counseling, social support mobilization, community outreach and follow-up.	High-intensity intervention: Less intensive intervention plus 5 additional home visits over a 30-month period. Emphasis on reducing barriers, increasing adherence, lifestyle modifications, family and social support. Addressed issues of health insurance and social/human service needs. Community-based: yes Home visits: yes	Low-intensity intervention: home visit with education, counseling, and referrals to promote access. Emphasis on adherence and monitoring. No control group without lay advisor intervention.
Hess, 2007	NonRCT *n* = 107 14 months	United States Low-middle income area of Dallas County (Urban) African-American Mean Age 51 years	Each barber underwent an initial four-hour training session that included BP measurement technique, BP interpretation, informed consent, and utilization of written project materials. In addition, barbers participated in monthly one-hour motivational booster sessions on intervention protocol fidelity.	BP measured with haircut, BP report card given with model story and patients were told to share BP report cards with their providers. If signed reports card returned- customer got a free haircut. Community based: yes Home visits: no	None
Balcazar, 2009	RCT *n* = 98 2 months	United States Urban Hispanic, 82% immigrants 52 y/o	Lay Hispanic community health workers (promotora) from surrounding communities. Trained for 4 days using the National Heart, Lung, and Blood Institute’s promotora curriculum, Salud Para Su Corazon (SPSC). Curriculum consists of educational materials designed to address CVD risk in the Hispanic community in the United States.	Group education: Nine-week program with educational modules in Spanish in 2-h sessions in weeks 1, 2, 3, and 8 for groups of 15–20. Modules focused on patient education, lifestyle modifications, and overcoming barriers, and a hypertension module with photonovela. Phone calls: Yes, during week 4 to 7 phone calls done to answer questions, schedule makeup classes, and discuss lifestyle changes discussed during educational modules Community based: Yes Home visits: No	Usual treatment, plus Spanish educational materials related to overall health provided in week 1
Jafar 2009	Cluster RCT *N* = 674 24 months	Pakistan Urban Asian Mean Age:53 years	Trained CHWs in behavior changing communications over 6 weeks.	First 90-min home health education visit session with all members of household, reinforcement visits of 30 min every 3 months for 2 yrs	Usual Care (No intervention)
Hayes, 2010	Non-RCT (Program description and adoption by peer leaders) 18 months	United States Rural and urban	27 peer leaders recruited from 15 veteran service organization posts. Initial 8-h training session followed by monthly/bimonthly 2-h meetings to introduce new health materials and review project activities. Topics covered hypertension, self-management and peer education/ motivation. 63 y/o (peer leaders) 78% peer leaders were males	Monthly group education: Educational materials, health scripts, BP check stations, and promotion of self-monitoring at routine veteran’s service organization meetings. Community based: No Home visits: No	No control arm
Truncali, 2010	NonRCT *n* = 244 6 months	United States Urban Ethnicity variable depending on site (Greek, Hispanic, Asian American, Jewish) 73 y/o	Community health workers recruited from members of senior center. Selection based on enthusiasm and availability. Trained for 12 h by health educators in BP measurement, record keeping, communication to participants.	Sessions every other week include checking and recording BP on program and participant record cards followed by information on BP status and recommended action steps Community based: Yes Home visits: no	No control arm
Victor, 2011	Cluster RCT *N* = 1,297 10-month	United States Urban, Dallas County African American 50-year-old	Barbers from black-owned barbershops with ≥95% black male clientele. Barbers trained, equipped, and paid to get consent, offer BP check, discuss a model story and give BP card to clientele	Community based: yes, Barber shops Baseline BP screening. Barbers continue BP monitoring and health and role model messages. If BP not at goal, barber discusses role model stories–real stories of other male customers modeling the desired changes in health behavior. Referral cards to physicians with incentive for returning physician signed card.	Baseline BP screenings followed by control group shops delivering standard pamphlets written by AHA. No BP monitoring.
Margolius, 2012	RCT *n* = 237 6 months	United States Urban 46% Hispanic and 35% Asian primarily (8% White and 11% Black also included) 60 y/o	Health coaches recruited from health center employees and volunteers with bachelor’s degrees. Trained for 16–20 h HTN, HTN medications, lifestyle modifications, adherence counseling.	High Intensity intervention of home BP monitoring, weekly health coaching, and home titration of BP medications Phone cells: weekly, to both study arms. Discuss well-being, adherence, BP values. Participants who reported elevated BP and adherence to medications could increase dose according to predetermined algorithm. Health worker advised physician to fax prescription and updated EHR. Community based: no Home visits: no	Low intervention: Home BP monitoring and weekly health coaching (no home titration). No control group without intervention.
Sanchez, 2014	NonRCT *n* = 96 2 months	United States Rural Hispanic 59 y/o	Lay Hispanic community health workers (promotora) from surrounding communities. Trained for 2 days using the National Heart, Lung, and Blood Institute’s promotora curriculum, Salud Para Su Corazon (SPSC). Curriculum consists of educational materials designed to address CVD risk in the Hispanic community in the US.	Group education: weekly session with 10–15 participants, 9-week curriculum. Plus, additional evidence-based mental health session and managing medicines session. Community based: Yes Home visits: Yes, reviewed and emphasized group education concepts.	No control arm
Ursua, 2014	Non RCT *N* = 88 4 months	United States Urban Asian Americans (Filipino) 53yo	Trained bilingual Filipino CHWs between 25 and 63 years of age. Previous CHW leadership and extensive community organizing experience. Number of training hours not reported	Group education: Yes for 90-min group education sessions delivered monthly, held in local library, community centers apartment buildings Phone: Yes, twice monthly phone calls Community based: yes Home visits: Yes, monthly as needed for individual visits at locations convenient for participants	No control arm
Dye, 2015	NonRCT *n* = 205 4 months	United States Rural 86% White 72 y/o	Community health workers recruited from community. Trained for 30 h in knowledge in modules. Required to score at least 80% on a post-training knowledge test and on a human subject’s protection test. Role plays to demonstrate proficiency in classroom instruction.	Group education: 8 core scripted modules plus 8 optional supplemental modules, each ~1.5 h long. Participants received notebook with interactive session activities, BP home monitors, cookbooks, pedometers, and relaxation CDs depending on modules completed. Participants counseled on developing personal action plans and use of a personal health diary. Following group training, health coaches met weekly with participants for an additional 7 weeks to implement core modules. Community based: yes Home visits: no	No control arm
Johnson, 2015	RCT *n* = 54 but only 37 completed the study 6 months	United States Urban African American 59 y/o	Experienced community health workers trained in International Society on Hypertension in Blacks (ISHIB) cardiovascular risk reduction toolkit. Training duration not mentioned.	Individual education session with community health worker based on ISHIB toolkit. Education included training in BP self-monitoring. Follow-up monthly visits included review of home BP measurements. Written reminder and 1 week phone call reminder for each appointment to confirm or reschedule. Community based: No (Physician offices) Home visits: no	Usual care, received similar assessments of outcome measures by CHWs for attention control
Dye, 2016	RCT *n* = 185 2 months	United States Rural White 65 y/o	Community health workers recruited who resembled demographic profile of the community. Training not reported. Required to achieve at least 80% on a knowledge test and demonstrate effective small group teaching skills.	Group education: 8 weekly small group classes, 90-min each, following scripted manual. Sessions focused on measuring BP, lifestyle modifications, relaxation using visualization CD, and long-term action plans. Focused on building self-efficacy. Participants provided with notebook with session notes, blood pressure monitor, pedometer, cookbook, relaxation CD. Community based: Yes Home visits: no	Usual care
Woods, 2016	Non-RCT *n* = 6 6 weeks	United States Urban free clinic 67% African American 52 y/o	CHWs recruited in partnership with a nonprofit agency. Trained during three 1-h sessions on HTN, BP monitoring, BP medications, and lifestyle modifications.	Community based: Yes Home visits: Yes, 3x, every 2 weeks for 6 weeks to review 2-week home BP records. CHWs discussed educational information based on AHA guidelines (HTN, medication, DASH diet, lifestyle modifications)	None
Goudge, 2018	RCT *n* = 3,413 18 months	South Africa Rural 55 y/o	Community health workers selected from the local community based on completion of secondary education. Trained by a local primary healthcare nurse with training qualification.	CHWs in clinics with usual care: appointment management, filing, health education on adherence and lifestyle, vitals measurements and prepackaging of medicines. Phone calls (or text): for appointment reminders Community based: no Home visits: no	Usual care
Neupane, 2018	Cluster RCT *n* = 1,638 12 months	Nepal Rural/urban: mixed Not reported 50 y/o	Female community health workers program includes 18 days basic training in primary healthcare training. Additional 5-day intensive training on hypertension for selected 46 CHWs for the study	Community based: Yes Home visits: Yes, every 4 months for a year (3 total visits). Provided lifestyle counseling and BP monitoring. Referred individuals with high BP to nearest health facility and followed for medication adherence.	
Ursua, 2018	RCT *n* = 240 8 months	United States Urban Asian 54 y/o	Community health workers were Filipino immigrants fluent in English and native languages. All had at minimum a bachelor’s degree and most lived in the same community as participants. Trained for 60 h in core competencies, HTN, lifestyle modifications.	Group education: yes, 4, 1x/monthly 90 min sessions consisting of health education using adult learning techniques and other culturally appropriate games. Individual visits between group sessions, 1x/month. Convenient locations including home, employer, community setting. Develop individual goals, remove barriers to access, promote adherence, provide referrals (including mental health or tobacco cessation). Phone: yes, as needed, to follow up with participants. Community based: yes Home visits: yes, as needed for individual visits.	One CHW delivered health education session was attended by control group.
Islam, 2018 (Beasley^d^, 2021) (37, 60)	RCT *n* = 187 6 months	United States Urban Asian Mean Age: 57 years (comorbid diabetes)	CHWs trained for 105 h in core competencies.	Group education: yes, monthly (5 total), 60 min health education sessions in English or South Asian language using culturally adapted curriculum. Phone calls: No 2 in-person 1-on-1 visits for action-planning and goal setting to improve diabetes management, conducted by CHWs in participants’ preferred language using standardized documentation tools	Usual care plus a single CHW-led 60 min group-based health education session in English or South Asian language using culturally adapted curriculum. Waitlist Control for 6 months
Gamage, 2019	RCT *n* = 1,736 3 months	India Rural Not reported 57 y/o	Community health workers recruited from accredited social health activists already serving the region. Trained for 5 days to deliver specific program.	Group education: every 2 weeks, for six 90 min sessions. Measured BP and educated participants on HTN and its management in local language. Education included medication adherence, lifestyle modifications. Community based: yes Home visits: no	Usual care
Joshi, 2019	RCT *n* = 3,261 12 months	India Rural Not reported 62 y/o	CHWs recruited from the community who had at minimum high school education. Trained for 4 weeks in survey methods, BP measurement, lifestyle counseling.	Community based: Yes Home visits: yes, every 2 months for 30–45 min, 6 total visits. Measured BP, assessed and reinforced medication adherence, delivered risk-reduction advice, links to physician for medication management.	Usual care defined as access to a clinic within 0 to 7 kilometers distance similar to Intervention group
Khetan, 2019	RCT *n* = 650 24 months	India Semi urban 52 y/o	CHWs recruited from community based on written test scores and 2 rounds of interviews. Trained for 7 days, 3 h/day (total 21 h) in 1–2-week blocks followed by 5 h supervised field work. Refresher training midway through study	Community based: Yes Home visits: yes, 1 h, every 2 months. Behavior change strategy focused on lifestyle, improving healthcare seeking, and addressing barriers to medication adherence. Communication in native language of patient using pictorial information.	Educational handout
Ojji, 2019	RCT *n* = 60 4 weeks	Nigeria Urban 43 y/o	Community health workers: training and selection not reported.	Two interventions (1): home blood pressure support (*n* = 20) and (2) community health worker support (*n* = 20). Community based: Yes In (2) community health worker support: Group education: yes, 4 education sessions over 4 weeks. Home visits: yes, 8 visits over 4 weeks for tailored counseling on health behaviors, adherence	Usual care (*n* = 20)
Poggio^c^, 2019	RCT *n* = 1,954 41 months	Argentina Urban 55 y/o	CHWs recruited from the primary care staff of public care centers. From the community, with similar ethnicity, language, SES, and life experiences. Trained for 2 days in motivational interviewing techniques, measuring BP, behavior change, adherence counseling, lifestyle modifications. Training followed by onsite field testing and certification.	CHW and text messaging intervention: Weekly, from web-based platform to participants and family members using a one-way outgoing system. Individualized to promote lifestyle changes and reminders for medication adherence. Community based: Yes Home visits: Yes, monthly for the first 6 months and every other month thereafter. Initial visit lasting 90 min with subsequent visits of 60 min. Family-based intervention to discuss general HTN knowledge at first visit. At subsequent visit, tailored counseling to participants and their families on adherence, home BP monitoring, and lifestyle modifications. Focus on goal setting, problem solving, social support, and motivation.	Usual care
Yi, 2019	NonRCT *n* = 146 6 months	United States, New York, Urban Asian 55 year old	Trained bilingual consultants recruited a team of Faith-based organization (FBO) volunteers. Participated in 2-day train the trainer program	Volunteers advertised programs. Worked through co-located senior center, every 2 weeks to quarterly, conducted free blood pressure screening, lifestyle counseling, weight management, coaching for clinical encounters.	No control group
Reininger, 2021	Non-RCT (RE-AIM Framework) *n* = 2,508 (12 locations) 5 years	United States Both urban and rural Hispanic 44 year old	CHWs recruited from local community and advocate for local needs. Trained to collect information from individuals on demographics, health habits, BMI, BP. Monthly training sessions throughout to ensure consistency and time for troubleshooting.	Community-wide campaign in 10 municipalities. CHWs lead the culturally tailored efforts of social support, risk factor screening, exercise groups, healthy cooking classes. Community based: Yes Group education: Yes Home visits: Yes	No control group
Schwalm, 2021	NonRCT *n* = 41 6 months	Canada Urban 69 y/o	CHWs and Firefighters: CHWs recruited from preexisting Community Health Center-associated individuals. 1-week CHW training Firefighters leveraged due to integration into the community via community fire check safety program with training in implementation of the intervention package.	CHWs and firefighters supported by tablets for data management and decision support, identified participants with poorly controlled hypertension, recommending evidence-based management strategy, supported lifestyle counseling Group education: no Individual education: yes. Each participant had a tailored intervention designed for their barriers, based on the 3 components above. Four total visits over 6 months. Online: No, but mobile health technology leveraged by CHWs. Community based: yes Home visits: no	None (compared to baseline)
Isiguzo, 2022	NonRCT *n* = 104 6 months	Nigeria Both urban and rural 57 y/o	Study participants recruited from selected religious centers. Role-model patients selected from enrolled patients. Must also have secondary education. Trained for 1 day in HTN, BP measurements, signs of uncontrolled HTN. CHWs helping with implementation trained for 3 days.	Peer-support adherence clubs led by role-model patients to motivate and facilitate medication adherence, BP monitoring. Once a month for 6 months in local community center. Group education: Yes, small groups (10–15) of patients with a role-model patient as facilitator Community based: yes Home visits: no	None (compared to baseline)
Samuel-Hodge, 2022	NonRCT *n* = 215 4 months	United States Rural 87% Non-Hispanic Black 57 y/o	CHWs hired from the community. Intensive 4-day training (20 h) on study protocols, intervention content, counseling principles, community referral resources, and data collection methods.	Carolina Heart Alliance Networking for Greater Equity (CHANGE) Program. Low intensity behavioral lifestyle interventions. Group education: no Home visits: Yes, 4 monthly counseling sessions in home (or at local venue selected by participant). Including goal setting, action planning, and resource referral. Phone calls: Yes, booster call after first three counseling visits Community based: Yes	None (compared to baseline)
Manavalan, 2022	NonRCT *n* = 14 4 weeks	Tanzania Urban Persons living with HIV (PLWH) 54.5 y/o	CHWs with a secondary level education & volunteer position with the local HIV advisory board. Two-week training by physician researcher on BP measurement techniques, risk factors, causes, symptoms, treatment, diagnosis, and prevention of hypertension. Review of intervention curriculum and mock intervention sessions.	Educational intervention based on the Health Belief Model delivered by CHW as part of HIV clinic appointments. Group education: No Phone calls: Yes (three in person sessions and two phone sessions). Remind patients of next appointment and to foster therapeutic relationship with CHW. Online: No Community-based: No Home visits: No	None. Comparison to baseline.
Suseela, 2022	RCT *n* = 1952 6 months	India Urban Asian 56.8 y/o	Pre-existing locally funded women’s self-help group (SHG) members were nominated by elected local representative to be trained to become peer educators. 1 per 20–30 households SHG members were provided a training module consisting of a facilitator’s guide, participant’s guide, presentations, and exercises on BP an anthropometry measurement. Modules included information on normal BP values, complications of HTN and diabetes, diet, and exercise recommendations as well as smoking cessation. SHGs were also trained on conducting patient support group meetings and guiding participants through goal setting. Trainings were conducted in groups of 15 and lasted for 21 h over 3 days. 6-h refresher was conducted 2 months later.	SHGs provided help with daily HTN management, encourage healthy behaviors via social and emotional support, and referral to primary healthcare centers. Group education: Yes. SHG members were assigned 1 to 20–30 households that met monthly. Each meeting lasted for an hour. Phone calls: No Online: No Community-based: Yes Home visits: Yes. SHG members visited participants at their homes to record their BPs, weight and provided counsel on diet, physical activity, and smoking cessation. Participants were also asked about their medication adherence and encouraged to take on healthy behaviors.	Usual care through clinics, private and public hospitals, and Urban Public Health Centers
Rimawi, 2022	Non-RCT *n* = 33 Patients graduated after 6 months at highest level of control	Refugees Palestine (Aida and Beit Jibrin camps, Bethlehem) Urban (Demographics provided only for patients with diabetes not hypertension)	CHW (Health for Palestine – H4P) aged 19–26 y/o Recruited by the Lajee Center via announcements in camp and via social media. CHWs were trained by IM physician and medical student using a guide from published CHW manuals. Training lasted for six weeks and consisted of lectures, role paying, hands-on training, and guided home visits with a physician or nurse.	Patients receive home visits by the CHWs based on the level of their disease control. An accompaniment approach, which encompasses medication and appointment adherence and support including transportation. Motivational interviewing to address the primary risk factors of diet, exercise, medication adherence, and smoking with integrated psychosocial counseling. Group education: No Phone calls: No Online: No Community-based: Yes Home visits: Yes	No matched controls for hypertension as BP data was not universally available.
Islam, 2023^d^	RCT *n* = 291 6 months	United States (New York City) Urban South Asian Americans 56.8 y/o	CHWs were chosen based on their representation of the patient population. They consisted of men and women, who spoke Bangla, Hindi, Punjabi and/or Urdu. They received standardized CHW core competency training after which they provided translation services to clinical staff, medication reminders, and appointment scheduling.	CHWs Group education: Yes. There were 5 monthly 60–90 min group education sessions. Phone calls: Yes, for follow up every two weeks. During follow-up, participants were guided to set goals for hypertension control (medication, activity, and nutrition). Some follow ups were completed in-person at primary care offices and community spaces. Online: No Community-based: Yes Home visits: No	Usual care plus a single initial CHW-led 60 min group-based health education
Kisigo, 2023	Non-RCT *n* = 50 1 year	Mwanza City, Tanzania Not Reported 61 y/o	Peer counselors were themselves patients with hypertension but a well-controlled blood pressure and receiving care at the outpatient clinic. Training duration: 2 weeks by medical doctors and social scientists.	The peer counselors provided five sessions on hypertension management over 3 months. Each session lasted about an hour except for the fourth telephone session lasting for 5-10 min Sessions focused on social connecting, medication adherence, lifestyle modifications including herbal options, following up in clinic and insurance coverage. Group education: No Phone calls: Yes, visit 4 was telephone call to check progress on medication adherence, going to clinic, and lifestyle changes. Online: No Community-based: Yes Home visits: Yes. First, third and fifth session were at patient’s home but patient could chose alternative location.	Historical controls receiving standard of care
Nelson, 2023	RCT *n* = 264 12 months	United States Veterans Washington, United States 60.6 y/o Black 28%	Seven peer health coaches were recruited to provide social support, assist with health education and goal setting and connect participants to community resources. Peer health coaches received 100 hours of comprehensive training in health coaching, and motivational interviewing by health psychologist and a clinician trained the health coaches in blood pressure monitor use.	Participants in this study received coaching for 12 months as well as educational materials, a blood pressure monitor, scale, pill organizer and healthy nutrition tools. Five required modules were completed during health coaching sessions: blood pressure, physical activity, nutrition, medication adherence, and communication with medical team or physician. Elevated BP was reported to primary care team. Group education: No Phone calls: Yes, for check-ins on progress related to goals. Online: No Community-based: Yes, Recruitment of both participants and coaches were based on the neighborhood. Home visits: Yes. 5 home visits and 5calls. Due to the COVID-19 pandemic, the protocol was changed to telephone only.	Usual care and same educational materials as intervention group. Elevated BP at enrollment or exit visit was reported to primary care team
Bush, 2023	Non-RCT *n* = 192 12 months	Northeast Texas, United States Rural 82% non-Hispanic	CHWs led several self-management blood pressure (SBMP) program workshop series to help improve participants’ health outcomes by providing heart health education. Information on CHW training was not reported.	The participants engaged in structured workshops, regular follow up, and were connected to community resources by the CHWs. Each workshop lasted for about an hour bi-weekly for 12 weeks. Group education: Yes Phone calls: No Online: No Community-based: Yes Home visits: No	None
Safford-Shikany, 2023	Cluster RCT *N* = 830^b^ 12 months	Black Belt AL and NC United States Rural Black 56% Age 58	Peer coaches provided support to patients as they made lifestyle changes to improve their blood pressure. This included goal planning, connection to community resources etc. Peer coaches received training and certification by staff members of the Southeastern Collaboration to Improve Blood Pressure Control (SEC). The coaches were members of the community just like the patients in the study. They also had chronic medical conditions but were not health professions and were not required to provide medical advice. They worked with patients for 12 months.	Assist engagement in hypertension self-management (including dietary changes and physical activity), to carry out the recommendations of the healthcare team (including taking medications and keeping appointments), to provide emotional support, and to link patients to the practice for care. Group education: No Phone calls: Yes, a one-on-one telephone delivered structured program intensive intervention phase of 8 weekly topic-focused sessions followed by monthly check-ins over the 12-month intervention period. Longer booster sessions were offered if BP control slipped after the intensive intervention phase. Online: No Community-based: Yes Home visits: No	Enhanced usual care - Each practice received a laptop computer, the freely available web-based patient education platform-Patient Activated Learning System (PALS), 25 home BP monitors and BP logs, and a binder of practice tips including flow sheets and an evidence-based BP titration algorithm designed for African Americans.
Thomas, 2023	Non-RCT *n* = not mentioned 8 participating barbers collected 236 BP readings 9 months	Barbershop clients BAME (Black, Asian, and Minority Ethnic) men Croydon, London	Barbers in an existing BAME barber network in south London, UK were recruited from 5 barbershops. Barbers were educated on offering BP checks to clients and providing education when needed. Trained online (1.5 h) and face-to-face (3 h)	Barbers provided BP healthcare advice and measured participants BP Group education: No Phone calls: No Online: No Community-based: Yes Home visits: No	None
Brewer, 2023	Non-RCT *n* = 16	Minneapolis-St Paul, MN African American 52.6 y/o	African American CHWs were hired for the study. CHWs monitored patient engagement on the app for self-monitoring and medication adherence. CHWs were trained by study PI on FAITH! App usage, content modules and NIH/NHLBI training manual. CHWs also received training for two months from the Association of Black Cardiologists Community Health Advocate Training (CHAT)	Patients utilized FAITH! Hypertension app and met with CHW weekly via telephone or zoom for 10 weeks. Group education: No Phone calls: Yes Online: Yes Community-based: No Home visits: No	None

^a^Only sample size of patients with hypertension is mentioned. ^b^If data on population/setting such as age or urban/rural location was not reported, we did not include the missing components in the study rows. ^c^For multi-arm studies, we limited the sample size intervention, and control group descriptions to groups where the only difference between groups was either a lay advisor intervention or a low-intensity versus a high intensity lay advisor intervention. ^c^original article for BP improvement included physician education component so was excluded but the current included article focused on lifestyle modifications supported by only CHWs with text messages. BP and hypertension control outcomes were not included in the review for the original article due to physician education component for medication management. ^d^Report of two RCTs with comorbid DM and HTN, included study information where CHW intervention was tailored for diabetes as the RCT with hypertension is separately included.

### 3.1 Study characteristics

Of the 41 articles meeting inclusion criteria, 22 were RCTs (16–37) and 19 were non-randomized studies including process assessments and matched cohort studies (38–56). Of the 22 RCTs, 13 RCTs were done in the US (16, 17, 21, 24, 26, 27, 29–31, 33, 35–37). Of the 19 non-randomized studies, 14 were done in the United States. Twenty-nine studies were from developed economies, and 12 were from developing economies. Studies mainly included racial/ethnic minority populations or were conducted in socioeconomically deprived areas of the country. Seven RCTs conducted in the United States included low-intensity lay advisor interventions in the control group (21, 24, 27, 30, 31, 35, 37). The participants’ mean age was 54 years and, in most studies, varied between 50 to 65 years.

Most studies mentioned lay advisors were matched demographically with study participants. The least intense interventions were outreach phone calls to promote access to care (29). Most intense intervention included monthly group education with home visits every other month with follow-up biweekly phone calls (35). Other than two studies, all lay advisor interventions were multicomponent. Seven studies compared low-intensity interventions with high-intensity lay advisor interventions. No studies specifically compared stratified or stepped care models of modifying lay advisor intervention intensity based on patient characteristics or hypertension control state with usual care.

### 3.2 Study quality

Supplementary Table S2 summarizes the assessment of risk of bias for individual randomized studies. All studies had at least one domain judged as unclear risk of bias and 18 studies had at least one domain, mainly blinding or intention to treat, regarded as high risk of bias. No studies had all domains regarded as low risk of bias. Dropout rates varied from 0 to 31% and 6 RCTs had dropout rates of >20% with higher dropouts from intervention groups.

### 3.3 Outcomes

Of RCTs where control groups did not receive any lay advisor interventions, nine reported systolic BP outcomes, eight reported diastolic BP outcomes and six reported hypertension control outcomes at baseline and end of study. See Table 2 for improvement in BP and hypertension control noted in all included RCTs and Table 3 for improvement in BP and hypertension control in included non-RCTs.

**Table 2:** Improvement in BP in RCTs^a^.

First authors	Reduction in SBP	Reduction in DBP	% Change in achievement of hypertension control (or proportion of patients achieving Hypertension control at end of study)
Krieger, 1999	−0.5 mm Hg in intervention vs. 0.5 mm Hg in control group	0 mm Hg in intervention vs. −0.1 mm Hg in control group	Not available
Morisky, 2002 (High-intensity intervention compared to low intensity intervention)	Not available	Not available	Hypertension control improved in the tracking group (by 7.8%, *p* < 0.01) and in the counseling group (by 13%, *p* < 0.01) compared to the control group (by 1.4%)
Levine, 2003 (High-intensity intervention compared to low intensity intervention)	−3.3 mm Hg in intensive intervention and − 5.6 mm Hg in less intensive intervention (*p* < 0.05)	−2.6 mm Hg in intensive intervention and − 3.8 mm Hg in less intensive intervention (*p* < 0.05)	Hypertension control improved by 20% in intensive intervention and by 16% in less intensive intervention
Balcazar, 2009	No statistically significant change in SBP/DBP between the intervention and control groups after adjusting for confounders	Not available	Hypertension control improved in intervention group (by 15%) and worsened in control group (−16%)
Jafar, 2009	−5.6 (95% CI −3.7 to −7.4)in CHW intervention group vs. −5.8 (95% CI −3.9 to −7.7) in control group(*p* = 0.89)	No statistically significant difference between intervention and control	Hypertension control improved in CHW intervention group by 23% and in control group by 27.3%, *p* = 0.003
Victor, 2011 (High-intensity intervention compared to low intensity intervention)	−7.8 mm Hg in intervention vs. −5.3 mm Hg in control group; absolute group difference of −2.5 mmHg [95% CI −5.3 to 0.3 mmHg] (*p* = 0.087)	−2.8 mm Hg in intervention vs. −1.9 mm Hg in control group; absolute group difference of −0.9 [95% CI −2.6 to 0.8] (*p* = 0.183)	Hypertension control improved in intervention group (by 19.9%) compared to control group (by 11.1%); absolute group difference 8.8% [95% CI 0.8 to 16.9%], *p* = 0.036
Margolius, 2012 (High-intensity intervention compared to low intensity intervention)	−23.9 mm Hg in home medication titration arm and − 19.3 mm Hg in no home titration; combined mean reduction of −21.8 mm Hg (*p* < 0.001)	−5.9 mm Hg in home medication titration arm and 5.4 mm Hg in no home titration; combined mean reduction of −5.7 mm Hg (*p* < 0.001)	Not available
Wallace-Johnson, 2015	−34.75 mm Hg [95% CI −46.55 to −22.95 mm Hg] in intervention group vs. −5.65 mm Hg [95% CI −12.84 to 1.54 mm Hg] in control group (*p* < 0.001)	−16.19 mm Hg [95% CI −24 to −8.39 mm Hg] in intervention group vs. −4.36 mm Hg [95% CI −8.26 to −0.46 mm Hg] in control group (*p* = 0.009)	At follow up, 83% of patients in intervention had hypertension control vs. 60% in usual care (*p* = 0.263)
Dye, 2016	Small (not reported) changes observed (*p* = 0.001) for both treatment and control groups	Small (not reported) changes observed (*p* = 0.018) for both treatment and control groups	Not available
Goudge, 2017	Not available	Not available	Hypertension control increased by 4.7% in intervention vs. 1% in control
Neupane, 2018	−4.9 mm Hg [95% CI −7.78 to −2.00 mmHg, *p* = 0.001] significant improvement in intervention participants compared to control group of hypertensive participants	−2.63 mm Hg [95% CI −4.59 to −0.67 mmHg, *p* = 0.008] significant improvement in intervention participants compared to control group of hypertensive participants	Not available
Ursua, 2018 (High-intensity intervention compared to low intensity intervention)	−20 mm Hg in intervention (*p* < 0.001) vs. −4.3 mm Hg in control group (*p* < 0.001) Intervention effect after adjustment for all covariates was −6.2 (*p* < 0.001)	−7.4 mm Hg in intervention (*p* < 0.001) vs. −0.2 mm Hg in control group (*p* = 0.829) Intervention effect after adjustment for all covariates was −2.8 (*p* < 0.001)	Hypertension control improved in intervention group (by 83.3%) compared to control (by 42.7%), *p* < 0.001. The adjusted odds of controlled BP for intervention was 3.2 times the odds for control [95% CI 1.9 to 5.4, *p* < 0.001]
Gamage,2019	−8.2 mm Hg [95% CI −10.0 to −6.3 mmHg] in intervention group vs. −2.1 mm Hg [95% CI −3.4 to −0.8 mmHg] in control group (*p* < 0.001)	−4.2 mm Hg [95% CI −5.3 to −3.1 mmHg] in intervention group vs. −2.2 mm Hg [95% CI −3.0 to −1.4 mmHg] in control group (*p* = 0.004)	Hypertension control improved in intervention group (by 20.3%) compared to control group (by 9.5%); odds ratio 1.6 [95% CI 1.2 to 2.1, *p* = 0.001]
Joshi, 2019	+0.4 mm Hg in intervention group vs. −0.5 mm Hg in control group over 12 months; no significant difference between groups	Not available	Not available
Khetan, 2019	−12.2 ± 19.5 mm Hg in intervention vs. −6.4 ± 26.1 mm Hg in control group; adjusted difference of −8.9 mm Hg [95% CI −3.5 to −14.4 mm Hg, *p* = 0.001]	−5.1 ± 13.5 mm Hg in intervention group vs. −3.0 ± 14.7 mm Hg in control group; adjusted difference of −2.1 mm Hg [95% CI −4.5 to 0.3, *p* = 0.09]	Hypertension control improved in the intervention group (by 17.6%) compared to control (by 8.6%), *p* = 0.23
Ojji, 2019	−31 mm Hg in intervention group vs. −21 mm Hg in control group, *p* = 0.02	−18 mm Hg in intervention group vs. −16 mm Hg in control group, *p* = 0.88	Not available
Poggio, 2019^b^	Not available	Not available	Not available
Islam, 2018 (Beasley, 2021)(60) (High-intensity intervention compared to low intensity intervention)	−5.7 mm Hg in intervention group (*p* < 0.001) vs. 0 mm Hg in control group (*p* = 0.98); adjusted odds ratio − 6.2 [95% CI −10.4 to −2.1]	−4.2 mm Hg in intervention group (*p* < 0.001) vs. 0 mm Hg in control group (*p* = 0.1); adjusted odds ratio − 4.0 [95% CI −6.3 to −1.7]	Hypertension control improved in the intervention group (by 24%, *p* < 0.001) compared to the control group (by 5.2%, *p* = 0.2); adjusted odds ratio 1.4 [95% CI 1.1 to 1.8]
Islam, 2023 (High-intensity intervention compared to low intensity intervention)	−6.8 mm Hg [95% CI −9.5 to −4.2 mmHg], significant improvement in intervention participants compared to control group participants	−4.7 mm Hg [95% CI −9.5 to −4.2 mmHg], significant improvement in intervention participants compared to control group participants	68.2% of the intervention group and 41.6% of the control group had controlled BP (*p* < 0.001)
Suseela, 2022	−4.1 mm Hg [95% CI −2.2 to −4.2 mmHg], significant improvement in intervention participants compared to control group participants	−1.5 mm Hg [95% CI −0.4 to −2.6 mmHg], significant improvement in intervention participants compared to control group participants	Not available
Nelson, 2023	No significant difference between intervention and control groups −2.05 [95%CI −7.00 to 2.55]	Not available	Not available
Safford, 2023	Results not reported	Results not reported	Results not reported

^a^*p*-values are not reported if they were not reported in the original article. ^b^Original article for BP improvement included physician education component so was excluded but the current included article focused on lifestyle modifications supported by only CHWs with text messages.

**Table 3:** Improvement in BP in non-RCTs^a,b^.

	^c^Reduction in SBP ± SD, mm Hg	^c^Reduction in DBP ± SD, mm Hg	% change in achievement of Hypertension control (or proportion of patients achieving Hypertension control at end of study)
Hovell, 1984 (Lay advisor intervention compared to Control group)	−10 mm Hg in intervention group (*p* < 0.05) vs. −2.4 mm Hg in control group. Follow up after intervention discontinued: −7 mm Hg in intervention vs. −13 mm Hg in control group.	−7 mm Hg in intervention group (*p* < 0.05) vs. −0.1 mm Hg in control group. Follow up after intervention discontinued: −7 mm Hg in intervention vs. 8 mm Hg in control group.	Not available
Hess, 2007	Not available	Not available	Hypertension control increased from mean 20% in those who did not participate to 51% in those with maximum intervention exposure. (*p* = 0.01)
Hayes, 2010	Not available	Not available	Not available
Truncali, 2010	−3.9 mm Hg in intervention participants with multiple visits [95% CI −7.6 to −0.1, *p* = 0.04]	Not available	Hypertension with controlled SBP increased by from 35 to 45% in intervention group (*p* = 0.16)
Sanchez, 2014	Not available	Not available	Not available
Ursua, 2014	−12.8 mm Hg reduction (*p* < 0.001)	−6.8 mm Hg reduction (*p* < 0.001)	Hypertension control increased by 27% from baseline to 4 months
Dye, 2015	−5.781 mm Hg in intervention group (*p* = 0.001)	−1.116 mm Hg in intervention group (*p* = 0.128)	Hypertension control increased by 11% in intervention group
Woods, 2016	Significant reduction in intervention group	Not available	Not available
Yi, 2019	−3.9 mm Hg in intervention group (*p* = 0.005)	−2.4 mm Hg in intervention group (*p* = 0.01)	Not available
Reninger, 2021 (High-intensity intervention compared to low intensity intervention)	−0.96% adjusted mean difference for intensive intervention compared to less intensive [95% CI −1.57 to −0.35, *p* = 0.002]	−1.61% adjusted mean difference for intensive intervention compared to less intensive [95% CI −2.42 to −0.81, *p* < 0.0001]	Low exposure (2–3 CHW visits) group was more likely than high exposure (4−5CHW visits) to recover to normal BP, OR 0.92 (*p* = 0.4) Hypertension control increased by 25% in the intervention group
Schwalm, 2021	−16.4 mm Hg (*p* < 0.01)	Not available	Hypertension control increased by 51% in the intervention group (*p* < 0.01)
Isiguzo, 2022	−13 ± 20.9 in intervention group (*p* < 0.0001)	−3.6 ± 12.1 in intervention group (*p* = 0.02)	28 of 65 patients changed from uncontrolled to controlled hypertension, 18 continued to have controlled hypertension
Samuel-Hodge, 2022	−2.9 ± 18.6 in intervention group (*p* = 0.03)	−2.1 ± 11.8 in intervention group (*p* = 0.007)	Hypertension control increased by 7% (*p* = 0.05) and DSB by 9% (*p* = 0.005) in intervention group
Manavalan, 2022	Median SBP reduced from 164 to 146 (*p* = 0.0029)	Median DBP reduced from 102 to 89 (*p* −0.0023)	Not available
Rimawi, 2022	−7.3 (95% CI −1.93 to −12.25, *p* = 0.009) in intervention group	−4.3 (95% CI −0.80 to −7.91, *p* = 0.018) in intervention group	Hypertension control increased by 58% after intervention
Kisigo, 2023	Not available	Not available	Combined rates of hospitalization and /or death were 18% in intervention cohort and 35% in historical controls.
Brewer, 2023	Mean pre-post SBP reduction −6.5 mm Hg (*p* = 0.15)	Mean pre-post DBP reduction –2.8 mm Hg (*p* = 0.78)	Proportion of patients with controlled Bp increased from 0 to 29%
Bush, 2023	Mean SBP reduced by 4.48 mm Hg (*p* < 0.05)	Significant reduction in DBP by –2.73 mm Hg	Not available
Thomas, 2023	Not available	Not available	Not available

^a^*p*-values are not reported if they were not reported in the original article. ^b^Other than Hovell, 1984 and Reninger, 2021, all non RCTs were pre- to post comparisons. ^c^Only included results for participants diagnosed with hypertension additional requirements. PRISMA checklist attached as a separate Supplementary Table.

#### 3.3.1 Effect on blood pressure outcomes

**Figure 2:**
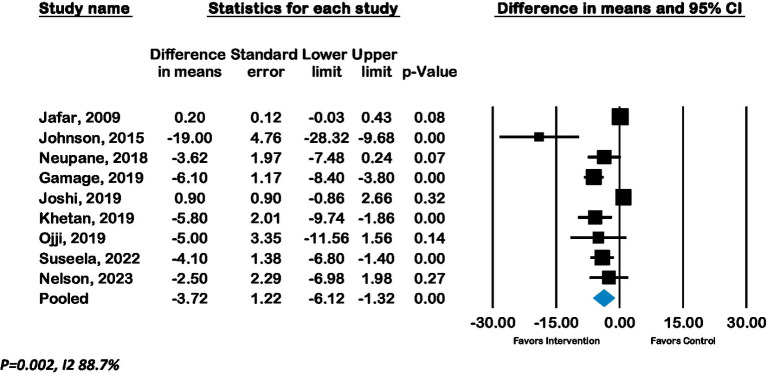
Forest plot of Systolic BP - Effect of lay advisor interventions on Systolic BP compared to control group.

**Figure 3:**
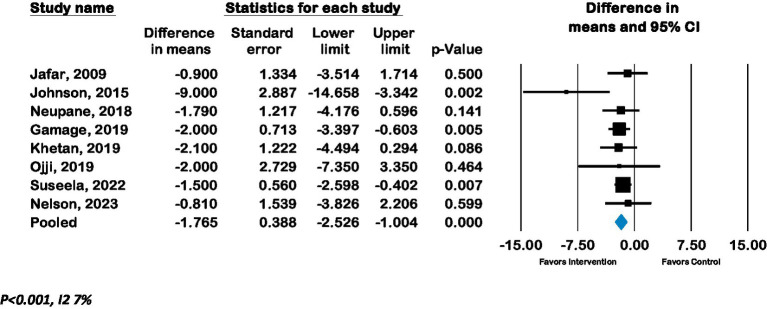
Forest plot of Diastolic BP - Effect of lay advisor interventions on Diastolic BP compared to control group.

The overall pooled effect of lay advisor interventions from nine RCTs (*n* = 4,220 participants) showed a mean improvement in systolic BP of −3.7 mmHg (CI –6.1 to –1.3; p 0.002, *I*^2^ 88%). (Figure 2) A sensitivity analysis where each study was removed had no significant impact on the results. (Supplementary Figure S1: Forest plot with each study removed) The pooled effect from eight RCTs (*n* = 3,056) of lay advisor interventions which measured diastolic BP showed an improvement of −1.8 mmHg (CI –2.5 to –1.0; *p* < 0.001, *I*^2^ 7%). (Figure 3) A sensitivity analysis where each study was removed had no significant impact on the results. (See Supplementary Figure S2).

#### 3.3.2 Effect on hypertension control outcomes

**Figure 4:**
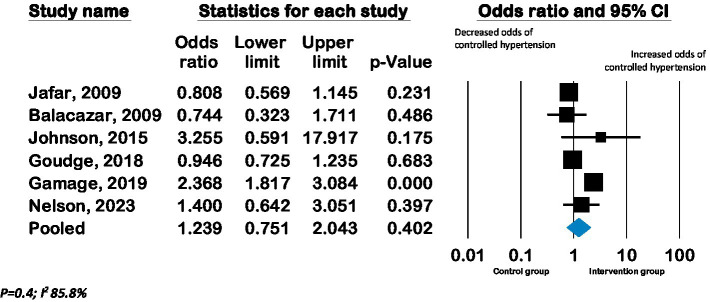
Forest plot of pooled effect on hypertension control - Effect of lay advisor interventions on hypertension control compared to control group.

Meta-analysis of six RCTs (*n* = 3,762) showed a pooled odds ratio of 1.2 (CI 0.75 to 2.0; *p* = 0.4, *I*^2^ 85.8%) for controlled hypertension with lay advisor interventions compared to the control group. (See Figure 4).

Subgroup analyses of studies grouped by presence of intention to treat did not show any significant differences in BP between groups. (Supplementary Figures S3, S4) There were not enough studies to conduct subgroup analyses of studies grouped by developed or developing country setting, and mode of interventions. The funnel plot and Eggers regression test (*p* = 0.008) indicate publication bias for systolic BP outcomes but not for diastolic BP or hypertension control outcomes. (Supplementary Figures S5, S6).

#### 3.3.3 Effect on systolic blood pressure, diastolic blood pressure, and hypertension control with high-intensity compared to low-intensity lay advisor interventions

**Figure 5:**
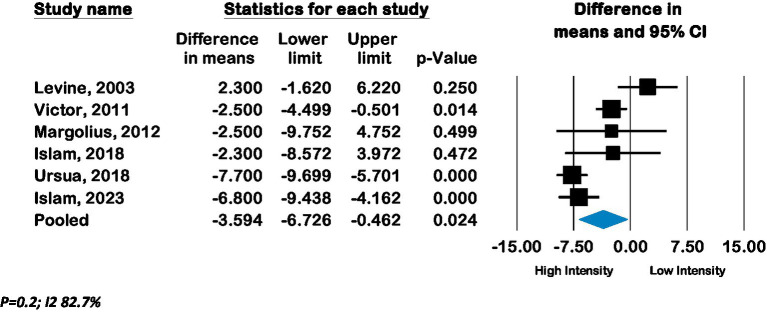
Forest plot of pooled effect of high intensity compared to low intensity interventions on Systolic BP.

**Figure 6:**
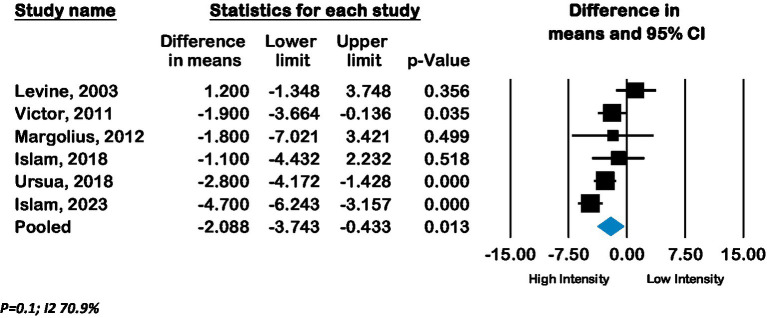
Forest plot of pooled effect of high intensity compared to low Intensity interventions on Diastolic BP.

Seven RCTs from the United States compared low-intensity interventions with high-intensity interventions, of which six reported BP outcomes. Pooled effect from these six RCTs (*n* = 2,644) showed a mean improvement in systolic BP of −3.6 mmHg (CI –6.7 to −0.46; *p* 0.02, *I*^2^ 82.7%) and in diastolic BP of −2.1 mmHg (CI –3.7 to −0.4; *p* 0.01, *I*^2^ 70.9%) in high-intensity lay advisor interventions compared to low-intensity interventions (See Figures 5, 6). The funnel plot and Eggers regression test (*p* = 0.4) did not indicate publication bias for these pooled BP outcomes. A sensitivity analysis where each study was removed showed reduced significance of results. (See Supplementary Figures S7, S8) Meta-analysis of seven RCTs (*n* = 3,277) showed a pooled odds ratio of 1.29 (CI 0.79 to 2.1; *p* = 0.3, *I*^2^ 90.79%) for controlled hypertension with high-intensity lay advisor interventions compared to the low-intensity lay advisor intervention group (Supplementary Figure S10). There were not enough studies in groups to conduct subgroup analyses for high intensity compared to low intensity interventions.

#### 3.3.4 RE-AIM criteria reporting In studies

There was no significant difference in the frequency of reporting of RE-AIM components between randomized and non-randomized studies other than qualitative assessments of efficacy, which were more frequently reported in nonRCTs. See Table 4 and Supplementary Table S1. One study specifically reported study results in RE-AIM format (45) and one recent study specifically reported reach and adoption of peer coaching intervention in primary care practices (33, 57).

**Table 4:** Publications reporting on RE-AIM elements.

Reach	Total (*n* = 41)	RCT (*n* = 22)	Non-RCT (*n* = 19)
Method to identify target population	100% (41)	100% (22)	100% (19)
Inclusion criteria	95% (39)	100% (22)	89% (17)
Exclusion criteria	53.6% (22)	68% (15)	36.8% (7)
Sample size	95% (39)	100% (22)	89% (17)
Participation rate	43.9%% (18)	50% (11)	36.8% (7)
Characteristics of both participation and non-participation	2% (1)	4.5% (1)	0% (0)
Representativeness of study population with target population	21.9% (9)	18% (4)	26% (5)
Efficacy/Effectiveness			
Measures/results (at shortest assessment)	97.6% (40)	95% (21)	100% (19)
Intent to treat (Only applicable to RCTs)		45% (10)	
Imputation procedures (specify)		18% (4)	
Quality of life measure	7% (3)	13.6% (3)	0% (0)
Effects at longest (specify time)	39% (16)	40.9% (9)	36.8% (7)
Percent attrition	78% (32)	86% (19)	68% (13)
Qualitative assessment	19.5% (8)	0 (0)	42% (8)
Adoption			
Method to identify target delivery agent	80.5% (33)	77.3% (17)	84% (16)
Level of expertise of delivery agent	68% (28)	72.7% (16)	63% (12)
Inclusion criteria	90% (26)	100% (22)	78.9% (15)
Rate	19.5% (8)	18% (4)	21% (4)
Characteristics of adoption/non-adoption	7% (3)	4.5% (1)	10.5% (2)
Qualitative assessment	24% (10)	13.6% (3)	36.8% (7)
Implementation			
Intervention type and intensity	100% (41)	100% (22)	100% (19)
Extent protocol delivered as intended (%)	34% (14)	22.7% (5)	47% (9)
Measures of cost	29% (12)	27% (6)	31.6% (6)
Qualitative assessment	29% (12)	22.7% (5)	42% (8)
Maintenance			
Was individual behavior assessed at least 6 months following the completion of the intervention?	14.6% (6)	9% (2)	21% (4)
Is the program still in place?	17% (7)	13.6% (3)	21% (4)
Was the program modified? Specify	14.6% (6)	9% (2)	21% (4)

##### 3.3.4.1 Reach

Six of the nine studies with sample sizes >1000 were conducted in developing countries (18–20, 23, 25, 34) and three were done in the United States (27, 31, 45). Participation rate varied from 2 to 98% with clinic-based recruitment showing higher participation rates compared to population-level recruitment. When reported, nonparticipating individuals had higher systolic BP or a lower proportion of their BP controlled at baseline, but this information was not reported in most studies (18, 29). Clinic-based recruitment showed higher participation rates as fewer patients needed to be approached in clinics (denominators were lower) with higher recruitment success (21).

##### 3.3.4.2 Efficacy/effectiveness reporting

Eight non-randomized studies reported qualitative assessments to understand outcomes (39, 41, 42, 50–53, 56); one of these was mainly a process evaluation of a sustained peer leader program for Veterans (39). One pragmatic randomized study included process evaluation in their protocol, but published article mentions challenges with balancing external validity and intervention assessments (19, 58). Few studies reported reasons for lay advisor withdrawals, which included personal reasons of health issues or relocation as well as an inability to perform certain required intervention tasks such as properly reading BP measurements (25, 39).

##### 3.3.4.3 Adoption

Method to identify the target delivery agent was reported mainly as the selection and nomination of volunteers with matching sociodemographic characteristics to participants. Two studies reported adding activities to a pre-existing program, but repeated visitor rate (43%) was only reported in one study (39, 44). One author of a randomized study shared a follow-up process evaluation using mixed methods assessment with surveys and focus group discussions (18, 59).

##### 3.3.4.4 Implementation

Four studies mention compensation for lay advisors reflecting the pay scale of the respective countries otherwise studies reported lay advisor compensation for completing study activities (18, 25, 28, 32). One study reported CHWs worked 40 to 60 h per month to care for 120 participants (28).

##### 3.3.4.5 Maintenance

Seven studies indicate the continued feasibility of maintaining intervention using already available infrastructure or public funding (25, 28, 32, 39, 44, 45, 60).

## 4 Discussion

We contribute to the literature by reporting a systematic review that evaluates the additional benefit of lay advisor interventions for hypertension outcomes where the lay advisor interventions are the sole additional intervention. We limited contamination by separating the analysis where the control group received any lay advisor intervention to inform this additional benefit and to inform differing intensity levels of interventions. We note improvement in blood pressure outcomes with added lay advisor interventions compared to usual care and with high-intensity interventions compared to low-intensity interventions in populations with lower socioeconomic states or racial/ethnic minority populations. Control groups and low-intensity interventions also showed BP improvements in most studies. No studies examined the impact of minimally burdensome lay advisor interventions with stepped-up care to high-intensity interventions in people with continued unmet needs.

Our results are similar to most reviews showing positive effects of CHWs on hypertension outcomes. Previous Community Preventive Services Task Force (CPSTF) found that team-based care with community health workers (CHWs) is effective for hypertension and cardiovascular disease prevention, but they did not look at the add-on benefit of CHWs (4). Team-based care and lay advisor interventions are difficult to translate into clinical care settings as reimbursement policies remain unclear for both, and staff time is limited (4, 5, 61). A recent review reported individual studies of CHW interventions in low-income and middle-income countries showed improved hypertension control (6). Most previous reviews focused on CHW interventions delivered by mainly government-trained CHWs or CHWs specifically recruited for the studies; lay advisors such as barbers and faith-based workers are infrequently included in previous reviews. We included a broader lay advisor definition and assessed the additional benefit of adopting lay advisors for hypertension care without adding burden to already overworked limited clinic staff within health systems. No previous reviews have compared the effectiveness of varying intensities of lay advisor interventions on hypertension outcomes. We strengthen the literature by showing the additional benefit of lay advisors interventions for improving hypertension outcomes and showing increasing effects with higher intensity interventions. Lastly, this is the first comprehensive systematic review of the state of lay advisor interventions for hypertension from internal and external validity perspectives using the RE-AIM framework. We also demonstrate areas for needed future research such as reporting on elements of the RE-AIM framework for the context of and validity of interventions as well as the need for examining stepped-up intervention intensity approaches for patients with continued uncontrolled hypertension which may help balance the intervention burden and limited healthcare resources.

### 4.1 RE-AIM assessment discussion

Similar to most literature, our RE-AIM assessments show frequent reporting of individual-level (Reach, Effectiveness) but insufficient reporting of organizational-level dimensions (Adoption, Implementation, and Maintenance) that affect the external validity of the interventions. Below we discuss each element of the RE-AIM Framework.

#### 4.1.1 Reach

Characteristics or contexts that interact with an individual’s willingness to participate may influence the potential of these interventions to improve health disparities, as most studies included socially disadvantaged populations.

##### 4.1.2 Effectiveness

Studies have rarely reported reasons for improvement in outcomes and characteristics of participants who may not benefit from these interventions or continue to have unmet needs. Assessments of multicomponent interventions to identify the least and most efficacious individual components are missing and may help tailor upscaling.

#### 4.1.3 Adoption

It is unclear what characteristics or contextual factors would encourage the uptake of a lay advisor role by individuals not already engaged in community-level leadership. Settings for lay advisor interventions were mostly predetermined with outside funding; hence, the characteristics of settings that otherwise may or may not participate are unclear.

#### 4.1.4 Implementation and maintenance

The time required for interventions’ key components and supervision needs to be quantified from the individual and organizational perspectives. Costs from a societal perspective or grant-funded compensation are frequently reported but may not be helpful for health systems with limited resources or budget margins.

### 4.2 Limitations

Our review has several limitations due to the way studies report information and limitations of meta-analyses. We strictly limited our review to studies with lay advisor-delivered intervention without additional health professional or research staff-delivered components. We did not want to combine two interventions with unclear reimbursement structures, and lay advisors are generally not yet part of core healthcare teams; however, lay advisors alone can provide support. Specifically, our exclusion of any physician education or training component limited a few key studies (62–65). We excluded these studies because physician-directed interventions may individually improve outcomes, and hypertension is routinely managed in time-restricted primary care clinic visits along with multiple concerns and health maintenance (66, 67). Secondly, as health systems may or may not be involved in community-level screenings but are typically held accountable for hypertension outcomes in their patient populations, we limited community-level screening studies to those reporting hypertension outcomes. Third, high heterogeneity was noted with diverse intervention components and intensity variations but planned meta-regression and subgroup analysis to explain the variation could not be done due to limited number of eligible studies; nevertheless, the increasing dose–response gradient with increasing intervention intensity supports directionality of the intervention effects. Literature syntheses can make sense of this heterogeneity if studies also report contextual factors affecting individual intervention component acceptability and efficacy.

### 4.3 Implications for future research

Our review has strong implications for future research. Reporting of most and least efficacious components of multicomponent interventions to tailor stepped upscaling of lay advisor interventions is needed. Studies mainly included adults representing the working population’s age where stepped-care models may be important to reducing intervention burden and balancing healthcare resource allocations. Tailoring lay advisor services within health systems that serve diverse patient populations has been understudied as most studies targeted socially disadvantaged population groups. Pragmatic trial designs such as hybrid effectiveness implementation trials may be helpful to evaluate not only how the intervention works but also how to successfully implement the interventions in diverse settings. Qualitative assessments of why and how the lay advisor interventions reach the targeted population, improve outcomes, and can be maintained are areas for future research. Future mixed methods assessments need to contribute to understanding the facilitators and barriers to engaging patients in the interventions, retaining a lay advisor workforce, and sustainability of the intervention at an individual and organizational level.

## 5 Conclusion

Add-on and high intensity lay advisor interventions may improve blood pressure outcomes in socially disadvantaged populations, but studied interventions are heterogeneous. Future studies need to identify the intervention’s most efficacious components and include assessments of stepped upscaling. Future research should focus on mixed methods assessments to identify explanatory processes for effectiveness and engagement at the individual, lay advisor, and setting levels to inform the real-world implementation of these interventions.

## Data availability statement

The original contributions presented in the study are included in the article/Supplementary material, further inquiries can be directed to the corresponding authors.

## Author contributions

SP: Conceptualization, Data curation, Formal analysis, Funding acquisition, Investigation, Methodology, Project administration, Resources, Software, Supervision, Validation, Visualization, Writing – original draft, Writing – review & editing. VB: Data curation, Formal analysis, Validation, Writing – original draft, Writing – review & editing. YY: Data curation, Formal analysis, Investigation, Methodology, Writing – original draft, Writing – review & editing. LB: Data curation, Validation, Writing – original draft. E-OU: Data curation, Validation, Writing – original draft. IT: Data curation, Validation, Writing – original draft. RS: Supervision, Writing – review & editing. KS: Methodology, Supervision, Writing – review & editing. SB: Methodology, Supervision, Visualization, Writing – review & editing.
