# Supplementary material for: Lay advisor interventions for hypertension outcomes: A Systematic Review, Meta-analysis and a RE-AIM evaluation
Source: Front Med (Lausanne). 2024 May 20;11:1305190. doi: 10.3389/fmed.2024.1305190 (PMC11144929; doi:10.3389/fmed.2024.1305190)
Supplement: Supplementary file 1 [file Data_Sheet_1.ZIP › Figure 1 PRISMA Flow Diagram.docx]

Figure 1: PRISMA Flow Diagram - Identification of studies via databases and registers

Records identified through database searching:

Ovid MEDLINE (n= 2539); CINAHL (n= 1228); Scopus (n=4760); Cochrane (n=560); PsycINFO with PsycARTICLES (n=1296); Sociological Abstracts (n=128); Clinical trials.gov (n=35)

Total: 10546

Records removed *before screening*:

Duplicate records (n= 3279)

## Identification

Records excluded:
(n = 7070)

Records screened
(n = 7267)

## Screening

Records excluded:

Not sole lay advisor or hypertension outcomes not reported separately for adults with hypertension (n = 156)

Full-text articles assessed for eligibility:
(n = 197)

Studies included in review

(n = 41)

## Included
