# Supplementary material for: Lay advisor interventions for hypertension outcomes: A Systematic Review, Meta-analysis and a RE-AIM evaluation
Source: Front Med (Lausanne). 2024 May 20;11:1305190. doi: 10.3389/fmed.2024.1305190 (PMC11144929; doi:10.3389/fmed.2024.1305190)
Supplement: Supplementary file 1 [file Data_Sheet_1.ZIP › Figure5and6_LowVsHighIntensity_BPForestPlots.docx]

**Figure 5: Forest Plot of Pooled effect of High Intensity compared to Low Intensity interventions on Systolic BP**

***P=0.2; I2 82.7%***

**Figure 6: Forest Plot of Pooled effect of High Intensity compared to Low Intensity interventions on Diastolic BP**

***P=0.1; I2 70.9%***
