# Supplementary material for: Lay advisor interventions for hypertension outcomes: A Systematic Review, Meta-analysis and a RE-AIM evaluation
Source: Front Med (Lausanne). 2024 May 20;11:1305190. doi: 10.3389/fmed.2024.1305190 (PMC11144929; doi:10.3389/fmed.2024.1305190)
Supplement: Supplementary file 1 [file Data_Sheet_1.ZIP › Revised_Figure 2and3_ForestPlot_BP.docx]

**Figure 2: FOREST PLOT OF SYSTOLIC BP - - Effect of Lay advisor interventions on Systolic BP compared to Control group**

***P=0.002, I2 88.7%***

**Figure 3: FOREST PLOT OF DIASTOLIC BP - Effect of Lay advisor interventions on Diastolic BP compared to Control group**

***P<0.001, I2 7%***
