# Supplementary material for: Lay advisor interventions for hypertension outcomes: A Systematic Review, Meta-analysis and a RE-AIM evaluation
Source: Front Med (Lausanne). 2024 May 20;11:1305190. doi: 10.3389/fmed.2024.1305190 (PMC11144929; doi:10.3389/fmed.2024.1305190)
Supplement: Supplementary file 1 [file Data_Sheet_1.ZIP › RevisedFigure 4_Hypertension Control.docx]

**Figure 4: Forest Plot of Pooled effect on Hypertension control - Effect of Lay advisor interventions on Hypertension Control compared to Control group**

Decreased odds of controlled hypertension

Increased odds of controlled hypertension

***P=0.4; I^2^ 85.8%***
