# Supplementary material for: Lay advisor interventions for hypertension outcomes: A Systematic Review, Meta-analysis and a RE-AIM evaluation
Source: Front Med (Lausanne). 2024 May 20;11:1305190. doi: 10.3389/fmed.2024.1305190 (PMC11144929; doi:10.3389/fmed.2024.1305190)
Supplement: Supplementary file 2 [file Data_Sheet_2.DOCX]

Supplementary Material

# Supplementary Data

Supplementary Material should be uploaded separately on submission. Please include any supplementary data, figures and/or tables.

Supplementary material is not typeset so please ensure that all information is clearly presented, the appropriate caption is included in the file and not in the manuscript, and that the style conforms to the rest of the article.

**Supplementary Method document 1: Search Strategy**

**Ovid Medline**

1 exp Hypertension/ or exp Antihypertensive Agents/

2 ("high blood pressur$" or "hypertens$").ti,ab.

3 "blood pressure".ti.

4 1 or 2 or 3

5 exp Community Health Workers/ or "Community Health Work$".ti,ab.

6 exp Volunteers/ or exp Peer Group/ or exp Barbering/

7 "Religion and Medicine"/ or exp Religion/ or "Religion and Science"/ or "Religion and Psychology"/

8 ("psychosocial care" or "self help group$" or "peer support$" or "therapeutic social club$" or "support group$" or "peer advoca$" or "peer mentor$" or "peer network$").ti,ab.

9 ("peer advis$" or "peer consultan$" or "peer counsel$" or "peer led" or "peer leader$" or "peer coach$" or "peer to peer$" or "peer tutor$" or "peer group$").ti,ab.

10 ("peer instruct$" or "peer facilitat$" or "lay led" or "lay run" or "lay expert$" or "lay worker$" or "lay person$" or "lay advisor$" or "lay consultan$" or "lay counsel$" or "lay leader$" or "lay educat$" or "lay tutor$").ti,ab.

11 (barber OR barbering OR beautician OR hairdresser OR hairstylist OR "beauty salon" OR "beauty culture" OR "beauty shop" OR "hair salon" OR salon OR stylist OR stylists OR salons OR cosmetologist).ti,ab.

12 (religion OR religious OR "religious beliefs" OR faith OR "prayer healing" OR "spiritual therap$" OR temple R church OR mosque OR synagogue OR "faith based" OR "faith placed" OR spiritual$).ti,ab.

13 ("lay instruct$" or "lay facilitat$" or "expert patient$" or "layperson$" or "voluntary worker$" or "volunteer worker$" or "trained volunteer$" or "volunteer aide$" or "user led$" or "mutual aid" or "promotora" or "culturally sensitive care" or "social support$").ti,ab.

14 5 or 6 or 7 or 8 or 9 or 10 or 11 or 12 or 13

15 4 and 14

16 limit 15 to (english language and yr="1981 -Current")

# Supplementary Figures and Tables

**SUPPLEMENTARY TABLE 1: EVALUATION DIMENSIONS AND MEASURES OF RE-AIM**

| Dimension | Indicator | Internal (1) or external validity indicator (2) | Description |
| --- | --- | --- | --- |
| **Reach** |  |  |  |
| The number, proportion, and representativeness of individuals who are willing to participate in a given intervention. | Methods to identify target population | 1 | Description of the process by which the target population was identified in the intervention |
|  | Inclusion criteria | 1 | Description of characteristics of the target population that were used to determine if a potential participant was eligible in the intervention |
|  | Exclusion criteria | 1 | Description of characteristics of the target population that prevent a potential participant from being eligible to participate |
|  | Sample size | 1 |  |
|  | Participation rate | 1 | Sample size divided by the target population denominator |
|  | Representativeness | 1,2 | Comparison of characteristics of the study participants in comparison to the target population |
| **Efficacy/Effectiveness** |  |  |  |
| The impact of an intervention on important outcomes, including potential negative effects. | Measures/results at least one follow-up | 1 | The study outcomes are measured at a time point after baseline |
|  | Intent-to-treat analysis utilized | 1 | Analyzing participants in trials in the groups to which they were randomized, regardless of whether they received or adhered to the allocated intervention |
|  | Quality of life or potential negative outcomes | 1 | Measuring acceptability and usability of the intervention in participants; evaluate unintended consequences that may result from the intervention |
|  | Attrition | 1 | The proportion that was lost of follow-up or dropped out of the intervention |
|  | Qualitative assessment | 1 | Efficacy or effectiveness was measured using qualitative methods |
| **Adoption** |  |  |  |
| The number, proportion, and characteristics of adopting settings and interventions agents | Description of intervention location | 1. 2 | Description of characteristics of the location of the intervention |
|  | Description of staff who delivered the program | 1, 2 | Description of characteristics of staff who delivered the intervention |
|  | Method to identify staff who delivered the intervention | 2 | Description of the process by which the staff was identified for participation in the study |
|  | Level of expertise of delivery agent | 2 | Training and educational background among those who delivered the intervention |
|  | Inclusion/exclusion criteria of delivery agent or setting | 2 | Description of the eligibility criteria of the setting/agent |
|  | Adoption rate of delivery agent or setting | 2 | The number of participating delivery settings or agents divided by the number of eligible and approached delivery settings or agents |
|  | Qualitative assessment | 2 | Qualitative evaluation of the settings in which intervention was adopted |
| **Implementation** |  |  |  |
| The extent to which the intervention delivered as intended (e.g., information on duration and frequency of intervention, fidelity to the intervention protocol, and cost including time and money) | Intervention duration and frequency | 1 | Length of the intervention (i.e. days, weeks, months and length of each intervention contact) and number of contacts with participants |
|  | Extent protocol delivered as intended (%) | 1 | Description of the fidelity to the intervention protocol |
|  | Measures of cost of implementation | 1, 2 | The costs including both money and time of delivery across all levels of implementation |
|  | Qualitative evaluation of implementation | 1 |  |
| **Maintenance** |  |  |  |
| The extent to which a participant maintains the change due to intervention and an intervention becomes institutionalized or part of the routine organization practices | Assessed outcomes ≥6 months post intervention | 2 | Description of follow-up outcome measures of individuals at some duration after intervention was terminated |
|  | Indicators of program level maintenance | 2 | Description of program continuation after completion of the research study |
|  | Measures of cost of maintenance | 2 | The ongoing cost of maintaining delivery across all levels of the intervention |
|  | Program adopted in other setting/populations | 2 | Description of the intervention being adopted beyond the original setting and population |

“Yes” or “no” were used to code the presence or absence of the RE-AIM indicators.

**SUPPLEMENTARY TABLE 2: COCHRANE’S RISK OF BIAS TABLE**

| Study Primary Author, Last Name | Randomization Technique of randomization | Blinding | Allocation concealment | Intention to treat | Missing data handling: | Dropout rate |
| --- | --- | --- | --- | --- | --- | --- |
| Krieger et al, 1999 | Yes | Unclear | Yes | No | Unclear | 26.1% |
| Donald Morisky 2002 | No | Unclear | Unclear | Yes | Unclear | 2% |
| Levine et al, 2003 | Yes | Unclear | Unclear | Yes | Yes | 13% |
| Balacazar et al, 2009 | Yes | Unclear | Unclear | Yes | Yes | 0% |
| Jafar, 2009 | Yes | Yes^a^ | Unclear | Yes | Yes | 22.7% |
| Victor et al, 2011 | Yes | Yes^a^ | Unclear | Yes | Yes | 21.2% |
| Margolius et al, 2012 | Yes | No | Yes | No | Unclear | 13.9% |
| Wallace Johnson, 2015 | Yes | No | Unclear | No | Unclear | 31.5% |
| Dye et al, 2016 | Yes | Unclear | Unclear | No | Unclear | 22% |
| Goudge, 2018 | Yes | Yes* | Yes | No | Unclear | 19.7% |
| Ursua, 2018 | Yes | No | Unclear | No | Unclear | 13.75% |
| Neupane, 2018 | Yes | No | Unclear | No | No | 10% |
| Islam 2018 (Beasley, 2021) | Yes | No | Unclear | No | No | 9% |
| Gamage 2019 | Yes | No | Unclear | Yes | Yes | 15% |
| Joshi, 2019 | Yes | Yes* | No | No | Unclear | 11.3% |
| Khetan 2019 | Yes | No | No | No | Unclear | 20% |
| Ojji, 2019 | Yes | Unclear | Unclear | Yes | Yes | 0% |
| Poggio 2019 | Yes | No | Unclear | Yes | No | 6.1% |
| Suseela, 2022 | Yes | No | Yes | Yes | Yes | 14.3% |
| Safford, 2023 | Yes | No | No | Unknown | Unknown | Ongoing |
| Nelson, 2023 | Yes | No | Yes | Yes | Yes | 15% |
| Islam, 2023 | Yes | No | No | No | Yes | 3% |

Footnotes:

Yes indicates low risk of bias; No indicates high risk of bias; ?Unclear risk of bias frequently due to not reported

^a^Outcome assessors blinded

## Supplementary Figures

**Supplementary Figure 1: Pooled effect for Systolic Blood Pressure with each study removed**

**Supplementary Figure 2 : Pooled effect for Diastolic Blood Pressure with each study removed**

**Supplementary Figure 3: Subgroup analyses of studies grouped by presence of intention to treat for Systolic BP**

***Between group p =0.5***

**Supplementary Figure 4: Subgroup analyses of studies grouped by presence of intention to treat for Diastolic BP**

**Supplementary Figure 5: Funnel Plot For Systolic Blood Pressure Outcomes:**

**Supplementary Figure 6: Funnel Plot For Diastolic Blood Pressure Outcomes**

**Supplementary Figure 7 : Pooled effect for Systolic Blood Pressure with each study removed for High Intensity interventions compared to Low Intensity interventions**

**Supplementary Figure 8: Pooled effect for Diastolic Blood Pressure with each study removed for High Intensity interventions compared to Low Intensity interventions**

**Supplementary Figure 9: Forest Plot of pooled odds for controlled hypertension for high intensity compared to low Intensity interventions**

Increased odds of controlled hypertension

Decreased odds of controlled hypertension

***P=0.3, I^2^ 90.79***

**
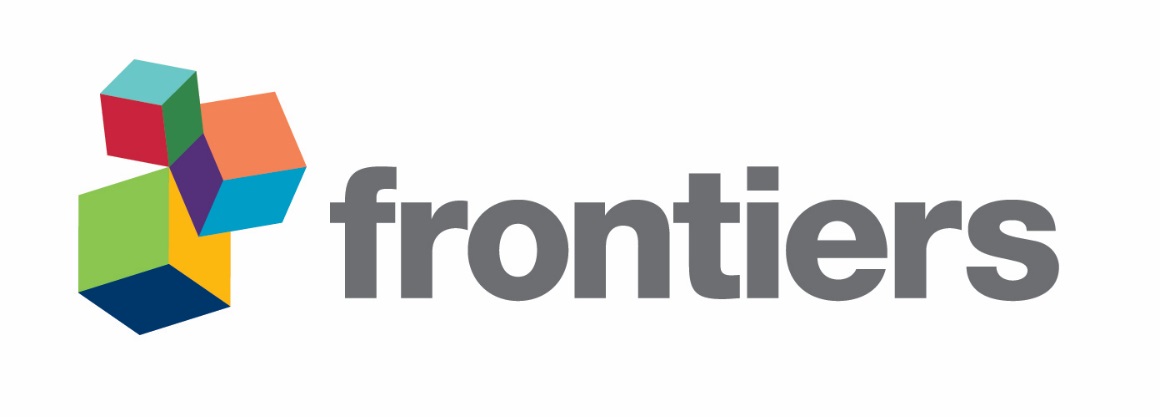
**

**Supplementary Figure 1.** The figure legends are required to have the same font as the main text, 12 point normal Times New Roman, single spaced. Please use a single paragraph for each legend and prepare the figures keeping in mind the PDF layout.
